# Effect of sodium-glucose transporter 2 inhibitors on sarcopenia in patients with type 2 diabetes mellitus: a systematic review and meta-analysis

**DOI:** 10.3389/fendo.2023.1203666

**Published:** 2023-07-03

**Authors:** Sha Zhang, Zhan Qi, Yidong Wang, Danfei Song, Deqiu Zhu

**Affiliations:** ^1^ Department of Pharmacy, Tongji Hospital, School of Medicine, Tongji University, Shanghai, China; ^2^ Department of Pharmacy, Ren Ji Hospital, School of Medicine, Shanghai Jiao Tong University, Shanghai, China

**Keywords:** sodium-glucose cotransporter 2 inhibitors, sarcopenia, type 2 diabetes mellitus, muscle mass, meta-analysis

## Abstract

**Objective:**

Sarcopenia has been recognized as the third category of disabling complications in patients with type 2 diabetes mellitus(T2DM), in addition to micro- and macrovascular complications. Sodium-glucose co-transporter 2 (SGLT2) inhibitors are innovative glucose-lowering treatments that have been shown to reduce body weight and enhance cardiovascular and renal outcomes. However, there is vigilance that SGLT2 inhibitors should be taken cautiously because they target skeletal muscle and may raise the risk of sarcopenia. Herein, we conducted a meta-analysis of randomized controlled trials to evaluate the effects of SGLT2 inhibitors on sarcopenia in patients with T2DM.

**Method:**

Relevant studies were obtained from PubMed, Embase, Medicine, Cochrane, and Web of Science databases to determine eligible studies until February 2023, without any language restrictions. A random effects model was utilized irrespective of heterogeneity, and the I^2^ statistic was used to evaluate study heterogeneity. The differences in results were measured using the weighted average difference (WMD) of the continuous data, along with a 95% confidence interval (CI).

**Results:**

A total of 25 randomized controlled trials with 2,286 participants were included. SGLT2 inhibitors significantly reduced weight-related changes and fat-related changes, including body weight(BW) (WMD= -2.74, 95% CI: -3.26 to -2.23, P<0.01), body mass index(BMI) (WMD= -0.72, 95% CI: -0.95 to -0.49, P<0.01), waist circumference(WC) (WMD= -1.60, 95% CI: -2.99 to -0.22, P=0.02), fat mass(FM)(WMD= -1.49, 95% CI: -2.18 to -0.80, P<0.01), percentage body fat(PBF) (WMD= -1.28, 95% CI: -1.83 to -0.74, P<0.01), visceral fat area(VFA)(WMD= -19.52, 95% CI: -25.90 to -13.14, P<0.01), subcutaneous fat area(SFA)(WMD= -19.11, 95% CI: -31.18 to -7.03, P=0.002), In terms of muscle-related changes, lean mass(LM)(WMD= -0.80, 95% CI: -1.43 to -0.16, P=0.01), and skeletal muscle mass(SMM) (WMD= -0.38, 95% CI: -0.65 to -0.10, P=0.007), skeletal muscle index(SMI) (WMD= -0.12, 95% CI: -0.22 to -0.02, P=0.02)were also significantly reduced. In addition, body water likewise decreased significantly (WMD=-0.96, 95% CI: -1.68 to -0.23, P=0.009).

**Conclusions:**

As one of the most widely used hypoglycemic, SGLT2 inhibitors have beneficial effects on FM and BW weight loss in T2DM, such as BW, BMI, WC, FM, PBF, VFA, and SFA. However, the negative influence on muscle mass paralleled the reduction in FM and BW, and the consequent increased risk of sarcopenia warrants high attention, especially as patients are already predisposed to physical frailty.

**Clinical Trial Registration:**

https://www.crd.york.ac.uk/prospero/#myprospero, identifier PROSPERO (No.CRD 42023396278).

## 1 Introduction

Sarcopenia is a syndrome that is common in elderly populations and is defined by age-related muscle mass loss, muscle strength decreased, and/or poor physical performance, all of which lead to functional decline, disability, frailty, and falls (1).

The European Working Group on Sarcopenia in Older People updated the clinically relevant definition and established an agreement on sarcopenia’s diagnostic standards in 2018, which encompass three main components: muscle quantity, muscle strength, and physical performance, and assessed by LM or SMM, assessed by hand grip strength, and assessed by gait speed or a short physical performance battery, respectively (2). Crucially, the guideline underscores that the reduction of SMM and LM represents a critical foundation for diagnosing sarcopenia in a clinical setting.

Moreover, Sarcopenia has been implicated as a serious consequence of T2DM (3). T2DM is a metabolic disorder characterized by insulin resistance, elevated advanced glycation end-products (AGEs), proinflammatory factors, and oxidative stress. These factors can disrupt normal cellular processes and result in microvascular and macrovascular complications, ultimately leading to cell death. As a result, individuals with T2DM may experience reductions in muscle mass, strength, and function, potentially precipitating the onset of sarcopenia (4). Kim et al (5) showed that patients with DM had a three times higher chance of developing sarcopenia than those without DM. Researchers and medics have been paying more attention to sarcopenia because of its serious impact on the quality of life of elderly patients and have therefore been recognized as the third category of disabling complications in patients with T2DM, in addition to micro- and macrovascular complications (6). DM is currently one of the most prevalent chronic non-communicable diseases globally, presently affects 537 million adults worldwide, and by 2045, it’s expected to affect 783 million people (7). It is widely recognized that hypoglycemic medications are pivotal in treating T2DM. However, glucose-lowering drugs that target skeletal muscle have the potential to impact SMM and function in T2DM patients.

SGLT2 inhibitors are gaining attention as novel oral hypoglycemic agents due to their distinct mechanism of decreasing proximal tubular glucose reabsorption and increasing urine glucose excretion, which has been shown to lower body weight and improves cardiovascular and renal outcomes (8, 9). Based on these important pharmacological effects, SGLT2 inhibitors are included in international authoritative diabetes guidelines and are widely used in clinical practice (10). However, there are cautions about using SGLT2 inhibitors, as they may raise the incidence of sarcopenia, especially in senior T2DM patients. Currently available studies published in this context have yielded inconclusive results. Therefore, it is necessary to conduct a comprehensive systematic review and meta-analysis of randomized controlled trials (RCTs) to assess the effects of SGLT2 inhibitors on sarcopenia in T2DM patients, to ensure medication safety and enhance the general health of elderly patients.

## 2 Materials and methods

### 2.1 Study design and search strategy

This meta-analysis was carried out following the Preferred Reporting Items for Systematic Reviews and Meta-Analyses (PRISMA) statement and was registered with PROSPERO (No. CRD 42023396278). We extensively examined the databases of PubMed, Embase, Medicine, Cochrane, and Web of Science for literature published before February 2023 using the following keywords: “Sodium-Glucose Transporter 2 Inhibitors”, “dapagliflozin”, “canagliflozin”, “empagliflozin”, “ipragliflozin”, “luseogliflozin”, “tofogliflozin”, “ertugliflflozin”, “sotagliflozin”, “sarcopenia”, “muscle mass”, “skeletal muscle”, “randomized controlled trials”. Manual searches were conducted on all found articles. To find additional material, we manually searched the references of relevant papers.

### 2.2 Study selection

We screened articles according to the following inclusion and exclusion criteria: Inclusion criteria: 1) All participants enrolled in the study were clinically diagnosed with T2DM and aged ≥18 years; 2) All chosen studies must be RCTs with SGLT2 inhibitors as the treatment and a placebo or another type of hypoglycemia medication as the control; 3) The outcomes should be sarcopenia relevant indicators, such as LM, SMM, SMI, gait speed, grip strength. Exclusion criteria: 1) studies with incomplete or inaccessible study data; 2) studies with unavailable primary outcome indicators; 3) duplicate literature studies; 4) non-RCT type research; and 5) experimental animal studies.

### 2.3 Data extraction and quality assessment

Study screening and data extraction from the relevant literature was carried out separately by two reviewers (ZS and WYD), when there were disagreements, a third researcher was consulted to reach a consensus. The following data were extracted:1) study characteristics (first author, publication year, country, intervention, sample size, follow-up time); 2) intervention characteristics (drug name, dose, duration of treatment, comparison, etc.); 3) primary outcome indicators (LM, SMM, SMI, gait speed, grip strength); and 4) secondary outcome indicators (BW, BMI, WC, FM, PBF, VFA and SFA.

According to the following seven criteria, the Cochrane Risk of Bias tool was used to evaluate the risk of bias: random sequence generation, allocation concealment, blinding of participants and personnel, blinding of outcome data, incomplete outcome data, selective reporting, and other biases. Each study was classified as a “low risk”, “high risk” or “unclear risk” of bias.

### 2.4 Statistical analysis

The weight mean difference (WMD) with 95%CI was used to quantify the pooled effects for continuous variable outcomes. All statistical analyses were performed using the RevMan5.4 software. The degree of heterogeneity in studies was evaluated using the I^2^ statistic. Studies with I^2^ statistics between 25% and 50% were regarded as having low heterogeneity, studies with I^2^ statistics between 50% and 75% as having moderate heterogeneity, and studies with I^2^ statistics above 75% as having high heterogeneity. A random-effects model was used in all studies, followed by either subgroup or sensitivity analysis to explicate the source of heterogeneity. Publication bias was assessed using funnel plots. P< 0.05 was considered statistically significant.

## 3 Results

### 3.1 Study selection

**Figure 1:**
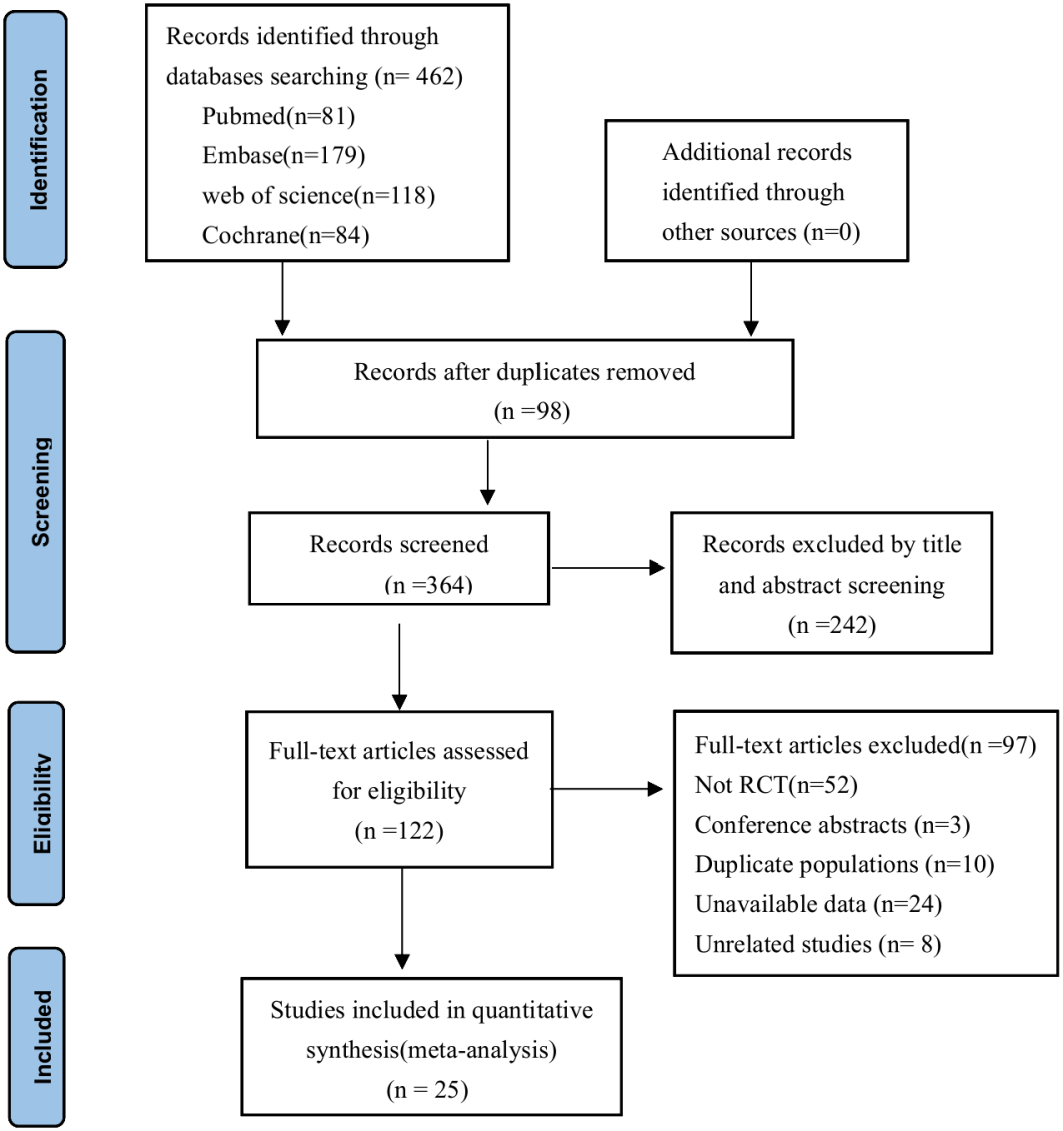
Flow chart of studies selected for the meta-analysis.

A total of 462 articles were selected based on the search strategy, of which 98 duplicate studies were removed using EndNote 20 software, 242 studies were excluded based on their titles and abstracts, and 122 studies were evaluated further for full-text examination. 25 studies total were eventually included in the meta-analysis. The detailed process is shown in **Figure 1**.

### 3.2 Studies characteristics and quality assessment

The included 25 research characteristics are shown in **Supplementary Table 1** (11–35). The intervention group consisted of a range of SGLT2 inhibitors, including dapagliflozin (ten studies), canagliflozin (five studies), empagliflozin (five studies), ipragliflozin (five studies), and tofogliflozin (one study). Meanwhile, the control groups received other hypoglycemic drugs, including metformin, glimepiride, pioglitazone, dipeptidyl peptidase-4 inhibitors (DPP-4 inhibitors), and Glucagon-like peptide-1 receptor agonists (GLP-1RAs). The follow-up period ranged from 8 to 104 weeks, with most studies lasting 24 weeks. Furthermore, all studies were high-quality parallel grouping studies according to the Cochrane Risk Bias Tool. As shown in **Supplementary Figure 1**.

### 3.3 Meta-analysis of outcomes

#### 3.3.1 Weight-related changes: BW, BMI, WC

**Figure 2:**
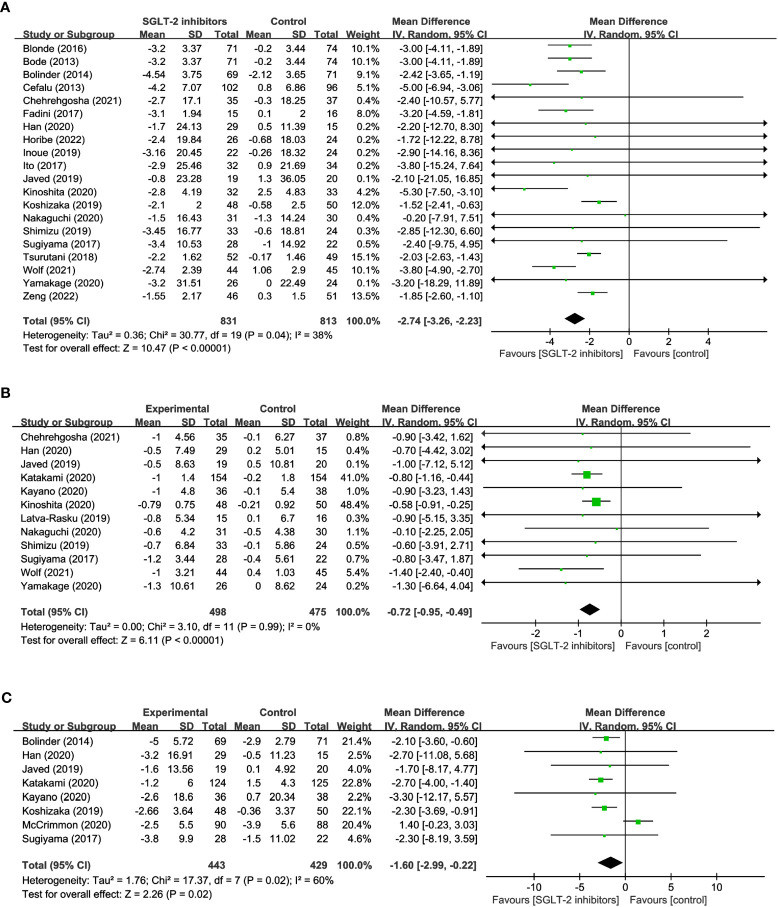
Forest plots of **(A)** BW, **(B)** BMI, and **(C)** WC.

20 studies reported on changes in BW in a total of 1,644 participants, of which 831 were treated with SGLT2 inhibitors and 813 were not. The meta-analysis showed that patients treated with SGLT2 inhibitors experienced a significant decrease in body weight compared to the control group (WMD= -2.74, 95% CI: -3.26 to -2.23, P<0.01) (**Figure 2A**), with low heterogeneity among the studies (I^2 =^ 38%). 12 studies reported BMI, comprising 498 SGLT2 inhibitor users and 475 non-users. The results suggest that treatment with SGLT2 inhibitors resulted in a statistically significant decrease in BMI when compared to other drugs (WMD= -0.72, 95% CI: -0.95 to -0.49, P<0.01) (**Figure 2B**), and no heterogeneity existed between the studies (I^2^ = 0%). 8 studies reported WC, with 443 using SGLT2 inhibitor and 429 non-use. In addition, when compared to the control group, patients in the SGLT2 inhibitor-treated group had a significantly smaller WC (WMD = -1.60, 95% CI: -2.99 to -0.22, P=0.02) (**Figure 2C**), however, there was considerable heterogeneity among the studies, (I^2^ = 60%). These results offer crucial information about the efficacy of SGLT2 inhibitors in reducing weight and can aid in the development of evidence-based interventions for obesity management.

#### 3.3.2 Fat-related changes: FM, PBF, VFA, SFA

**Figure 3:**
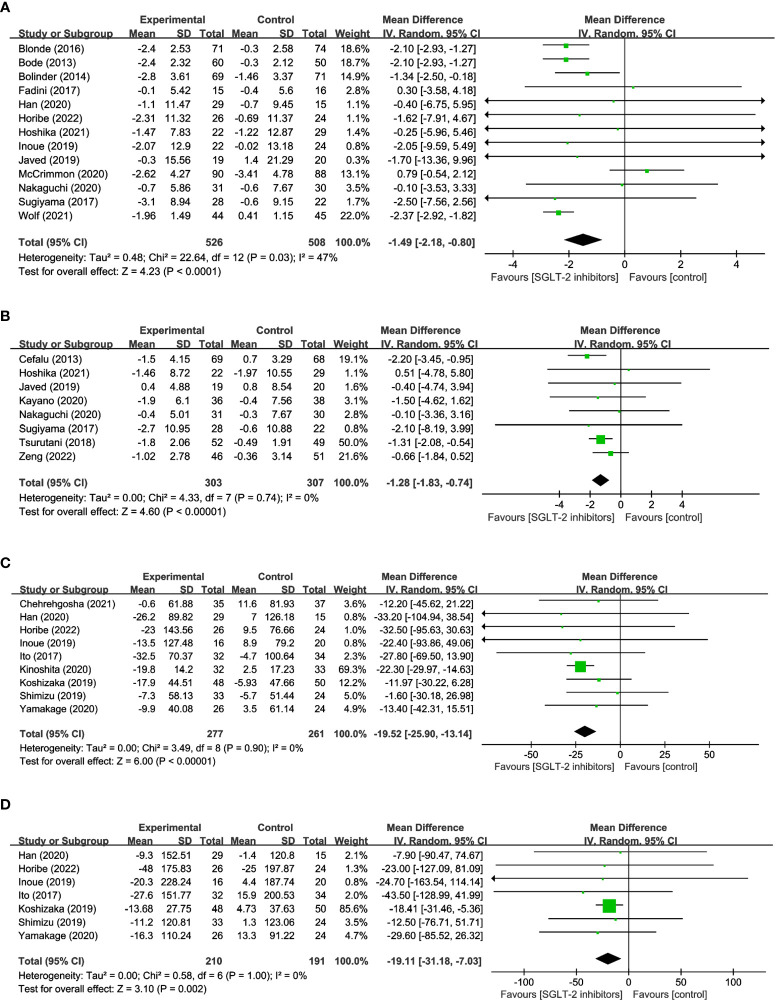
Forest plots of **(A)** FM, **(B)** PBF, **(C)** VFA, and **(D)** SFA.

13 studies involving 1,034 participants were analyzed in FM, with 526 using SGLT2 inhibitors and 508 non-users. And the results indicated that SGLT2 inhibitors significantly reduced FM when compared to other antihyperglycemic drugs (WMD = -1.49, 95% CI: -2.18 to -0.80, P<0.01) (**Figure 3A**), albeit with moderate heterogeneity (I^2^ = 47%). 8 studies explored the impact of SGLT2 inhibitors on PBF in 610 participants. Results suggested that SGLT2 inhibitors dramatically decreased PBF in comparison to the control group (WMD = -1.28, 95% CI: -1.83 to -0.74, P<0.01) (**Figure 3B**), with no observed heterogeneity (I^2^ = 0%). 9 studies were identified that reported measuring VFA in a total of 488 individuals, with 227 using SGLT2 inhibitors and 261 non-users. The findings indicated that SGLT2 inhibitors greatly decreased VFA compared to other anti-glycemic drugs (WMD= -19.52, 95% CI: -25.90 to -13.14, P<0.01) (**Figure 3C**), with no heterogeneity among the studies (I^2^ = 0%). Besides, the effects of SGLT2 inhibitors on SFA were evaluated in 7 trials in 210 SGLT2 inhibitor users and 191 non-users. The outcomes additionally demonstrated that SGLT2 inhibitors markedly decreased SFA more than the control group (WMD = -19.11, 95% CI: -31.18 to -7.03, P=0.002) (**Figure 3D**), with no heterogeneity between the studies (I^2^ = 0%). The aforementioned findings indicate that SGLT2 inhibitors may be a more efficient alternative for managing fat-related alterations in people with hyperglycemia, as they have demonstrated efficacy in reducing FM, PBF, VFA, and SFA. These results suggest that SGLT2 inhibitors could be a viable option for managing metabolic complications associated with hyperglycemia-related conditions.

#### 3.3.3 Muscle-related changes: LM, SMM, SMI

**Figure 4:**
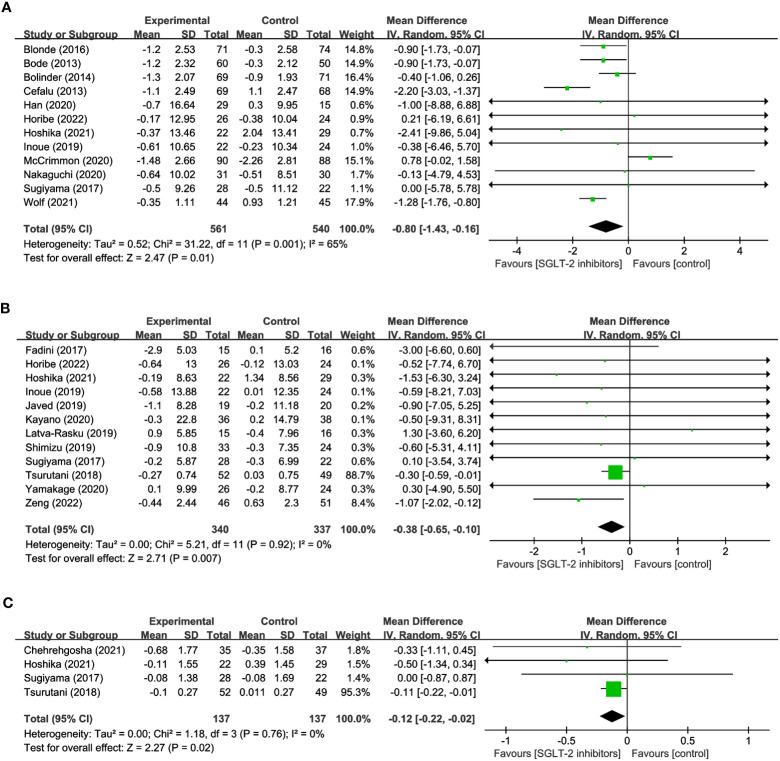
Forest plots of **(A)** LM, **(B)** SMM, and **(C)** SMI.

12 studies were conducted to assess the effects of SGLT2 inhibitors on LM using DXA involved in 1,101 participants. The overall analysis indicated a significant reduction in LM with SGLT2 inhibitors compared to other antihyperglycemic drugs (WMD= -0.80, 95% CI: -1.43 to -0.16, P=0.01) (**Figure 4A**), with a moderate degree of heterogeneity observed among the studies (I^2^ = 65%). Similarly, 12 studies involving 340 SGLT2 inhibitor users and 337 non-users were evaluated for SMM, and the results revealed a significant reduction in SMM with SGLT2 inhibitors compared to other antihyperglycemic drugs (WMD = -0.38, 95% CI: -0.65 to -0.10, P=0.007) (**Figure 4B**), with no heterogeneity observed (I^2^ = 0). Furthermore, 4 studies including 137 SGLT2 inhibitor users and 137 non-users were analyzed to assess SMI using BIA, and the results indicated a significant reduction in SMI with SGLT2 inhibitors compared to other antihyperglycemic drugs (WMD= -0.12, 95% CI: -0.22 to -0.02, P=0.02) (**Figure 4C**), and no heterogeneity was found among the studies (I^2^ = 0). These findings imply that SGLT2 inhibitors may negatively impact LM, SMM, and SMI, and should be considered when developing treatment plans for individuals with hyperglycemia-related conditions.

#### 3.3.4 Fluid-related changes: body water

**Figure 5:**
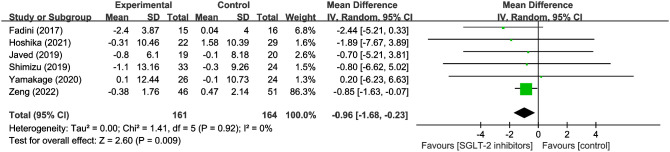
Forest plot of body water.

6 studies evaluated s body water in 161 SGLT2 inhibitor users and 164 non-users. The results revealed a significant reduction in body water with SGLT2 inhibitors compared to other hypoglycemic drugs (WMD = -0.96, 95% CI: -1.68 to -0.23, P=0.009) (**Figure 5**), with no heterogeneity observed (I^2^ = 0). The results imply that it is critical to take into account the potential loss of body fluids when using SGLT2 inhibitors.

### 3.4 Sensitivity analysis and subgroup analysis

**Figure 6:**
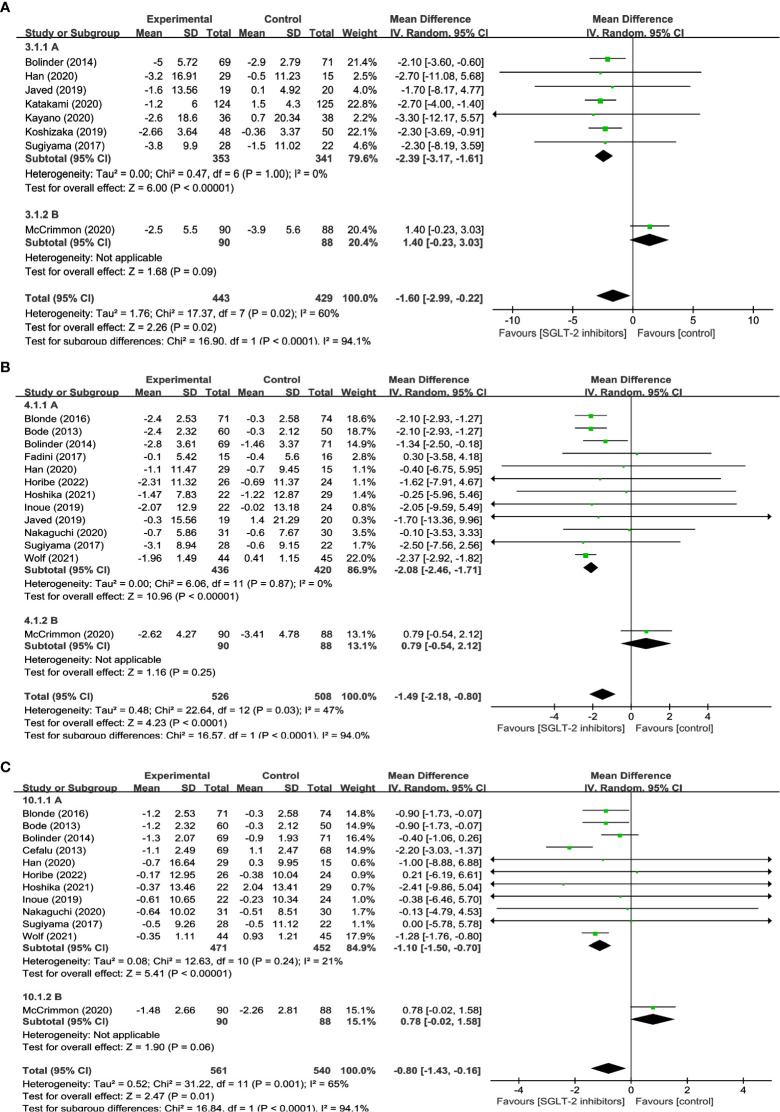
Subgroup analysis of **(A)** WC, **(B)** FM, and **(C)** LM.

Sensitivity analyses were carried out to identify the causes of heterogeneity. When McCrimmon’s study was removed, the heterogeneity in terms of WC, FM, and LM was significantly decreased. The findings indicated that SGLT2 inhibitors significantly reduced WC (WMD= -2.39, 95% CI: -3.17, -1.61, P<0.01), FM (WMD = -2.08, 95% CI: -2.46, -1.71, P<0.01), and LM (WMD = -1.10, 95% CI: -1.50, -0.70, P<0.01), with lower effects than GLP-1RAs, but the differences were not statistically significant WC (WMD = 1.40, 95% CI: -0.23, 3.03, P=0.09) (**Figure 6A**), FM (WMD= 0.79,95% CI: -0.54, 2.12, P=0.25) (**Figure 6B**), and LM (WMD = 0.78, 95% CI: -0.02, 1.58, P=0.06) (**Figure 6C**). Due to low heterogeneity, other outcomes including BW, BMI, SMM, SMI, VFA, SFA, PBF, and Body water were not tested further.

### 3.5 Publication bias

**Figure 7:**
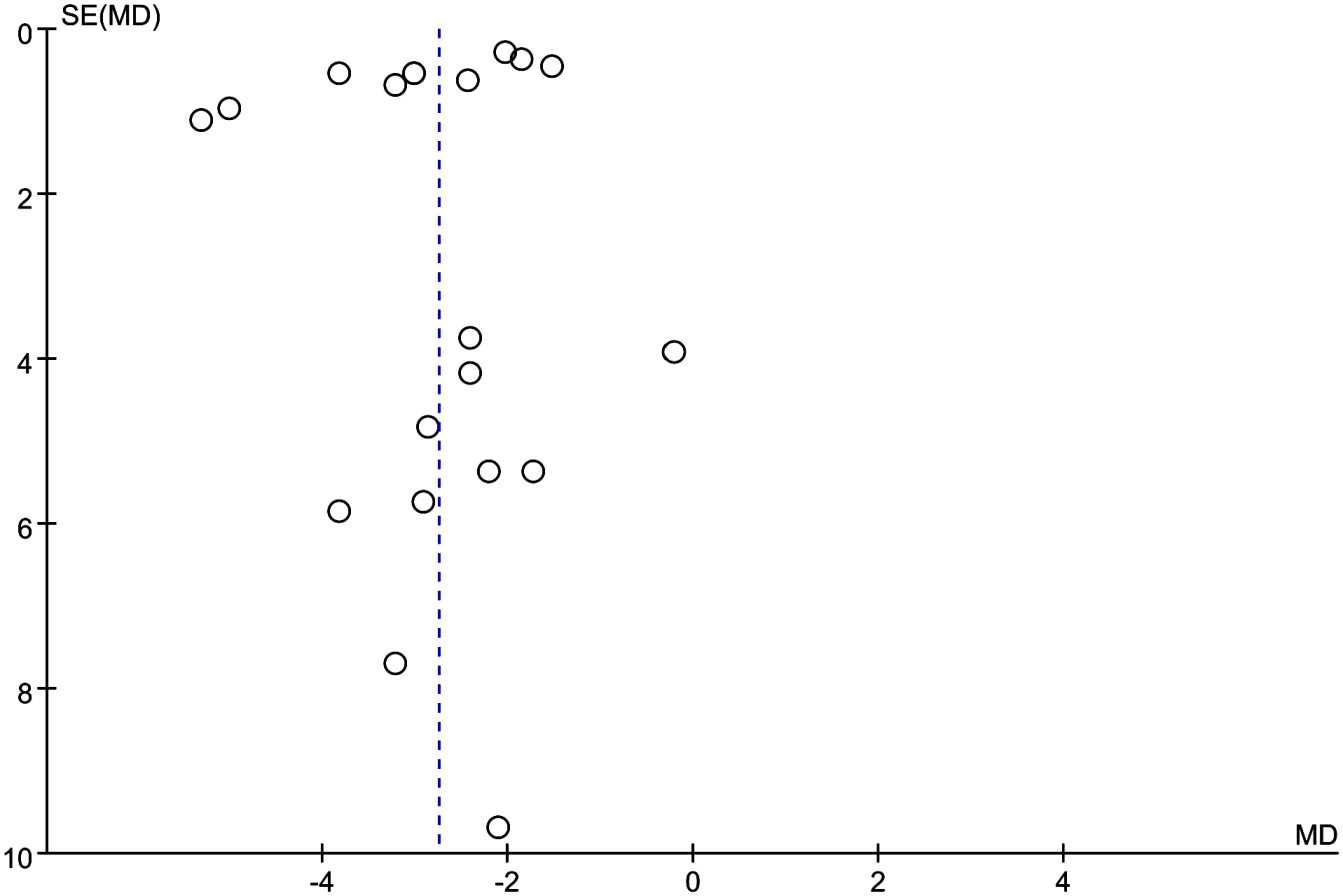
Funnel plot of BW.

Publication bias was assessed using funnel plots (**Figure 7**), which showed that the scatter points pertaining to each study were mainly dispersed on the midline or largely symmetrically distributed.

## 4 Discussion

We demonstrated that SGLT-2 inhibitors may increase the risk of sarcopenia in diabetic patients. As we found, in addition to greatly lowering BW and FM in T2DM patients, SGLT2 inhibitors also significantly lowered LM, SMM, and SMI and consequently increased the risk of sarcopenia.

There exist multiple bidirectional relationships between T2DM and sarcopenia, whereby the presence of one condition may elevate the likelihood of developing the other and make it a significant public health concern (6). T2DM represents a state of accelerated metabolic aging, and a portion of its associated frailty risk may stem from an escalated decline in muscle mass and function. Decrease in muscle mass and function, which are linked to reduced muscle strength and endurance, also lead to a higher risk of falls and physical frailty (36, 37). Consequently, Elderly diabetics with combined sarcopenia experience more pronounced metabolic abnormalities, suboptimal nutritional status, and increased susceptibility to developing osteoporosis and falls, which decrease quality of life and increase mortality (38). Hence, the<<Guideline for the management of diabetes mellitus in the elderly in China(2021 edition)>>recommends healthcare practitioners promptly evaluate sarcopenia in all older patients with diabetes (39).

SGLT2 inhibitors are novel antihyperglycemic drugs that decrease proximal tubular glucose reabsorption, which raises urine sugar excretion and lowers blood glucose levels. These drugs have received high attention due to their glucose-dependent mechanisms of action, and pose a low risk of hypoglycemia, particularly when used without insulin or sulphonylureas (40). Importantly, SGLT2 inhibitors also reduce body weight, blood pressure, urine protein, and uric acid, and improve adipocyte dysfunction in visceral adipose tissue, resulting in lower leptin, vastatins, fibrinogen activator inhibitor-1, and higher lipocalin levels, effectively promoting lipolysis and reducing visceral fat, thereby achieving a cardiovascular benefit (41, 42). To sum up, SGLT2 inhibitors are recommended by the guidelines for the following chronic diseases: diabetes, obesity, cardiovascular disease, and kidney disease. However, Sarcopenia may be a major concern and the most significant barrier to SGLT2 inhibitor use (43). SGLT2 inhibitor-promoted activation of gluconeogenesis resulting from the decrease in insulin levels and increase in glucagon levels, which may lead to lipolysis in adipose tissue and proteolysis in skeletal muscle, could supply amino acids to the liver and potentially contribute to sarcopenia (44). Clinical studies of sarcopenia caused by SGLT2 inhibitors in T2DM patients have been published. Typically, Nagai Y. et al. found that ipragliflozin reduced the weight of FM and the LM (45). Conversely, other studies have shown that dapagliflozin dramatically decreased FM but had no impact on lean tissue mass (46). Currently, it is unknown whether SGLT2 inhibitors exert a deleterious impact on sarcopenia. Thus, it is crucial to evaluate the effect of SGLT2 inhibitors on sarcopenia in T2DM patients. Previous studies have indicated that sarcopenia involves a complex interplay of metabolic dysregulation, insulin resistance, fat infiltration, fibrosis, and neural activity. Interestingly, there is considerable overlap in the etiology of sarcopenia, obesity, and T2DM, with obesity-related insulin resistance being one of the primary pathogenic mechanisms underlying T2DM and potentially contributing to sarcopenia’s underlying mechanisms (47, 48). Parallel to insulin resistance, fat infiltration contributes significantly to impairments in muscle quality and function. Thus, weight loss is a crucial goal in the management of obesity-associated chronic metabolic diseases, including T2DM, and pharmacological interventions that promote weight loss are attractive and feasible. Previous research has shown that SGLT2 inhibitors help with both BW and FM weight loss, with Kawata T et al. estimating that body fat accounts for 50% to 75% of SGLT2-induced weight loss (49). While BMI and WC represent quick, convenient, and reliable measures of obesity, they are relatively broad indicators that do not account for fat distribution and thus have limited helpfulness in predicting the risk of T2DM and sarcopenia (50). To further our understanding of sarcopenia in T2DM patients, this meta-analysis employs multiple body composition measurements including FM, BFM, VAT, and SAT, and confirmed the role of SGLT2 inhibitors in reducing BW, BMI, WC, FM, and BFM, which agreed with the results of earlier researches. In addition to reducing FM, SGLT2 inhibitors decrease VAT and SAT. Although the exact mechanism by which SGLT2 inhibitors reduce adipose tissue is unknown, some studies have shown that they promote a metabolic switch from carbohydrate oxidation to fatty acid oxidation, boosting the liver’s and adipose tissues’ fatty acid oxidation to potentially prevent lipid accumulation (43). Furthermore, they facilitate energy loss through a sustained increase in glucose excretion in urine, which may boost b-oxidation in the liver and visceral fat, enhance liver fat metabolism, and decrease VAT and SAT levels (51). In addition, our meta-analysis revealed that SGLT2 inhibitors dramatically enhanced body water loss in comparison to other conventional glucose-lowering treatments, which prior meta-analyses had not mentioned (52, 53). It could be explained because the unique hypoglycemic mechanism of SGLT2 inhibitors through urinary glucose excretion takes away some water while excreting sugar, which may contribute to weight loss. However, it is worth mentioning that studies also have reported instances of ketosis and euglycemic ketoacidosis caused by dehydration and insulinogenic during the use of SGLT2 inhibitors (54). As a potentially severe adverse reaction, ketosis demands our utmost attention when using SGLT2 inhibitors.

Some research has shown that using SGLT2 inhibitors reduces LM along with weight reduction. Outstandingly, Bolinder J et al. found that approximately 2/3 of the weight loss brought on by SGLT2 inhibitors was responsible for a decrease in FM, while a decrease in LM was responsible for the remaining 1/3 (55). Our meta-analysis confirmed that SGLT2 inhibitors considerably reduced both LM and SMM when compared to other traditional hypoglycemic therapies, which was in line with the conclusions of previous studies. In this meta-analysis, we analyzed 12 studies that reported changes in BW and LM, and the loss of LM accounted for between 10% and 40% of the BW lost, with an average of around 30%, roughly consistent with Bolinder J’s conclusion. Considering the subtle differences between LM and SMM, LM was measured by Dual-energy x-ray absorptiometry, which includes muscle, organs, and body water, whereas SMM was measured by bioelectrical impedance analysis. Our meta-analysis further assessed the BW due to SMM, interestingly, it found that SMM accounted for around 1/3 of weight loss, and the reduction in SMM accounts for a non-negligible proportion of the weight loss with SGLT2 inhibitors. Furthermore, we also verified the benefits of SGLT2 inhibitors in lowering SMI that were not previously included in meta-analyses (52). It is worth mentioning that SMI, which is defined as SMM/height^2^ or SMM/BMI, is an underappreciated evaluation metric in sarcopenia research. As stated previously, although total body SMM or LM measurements can be used to estimate muscle quantity, the relationship between muscle mass and body size is crucial; people with larger frames often have greater muscle mass, so SMM can be adjusted for body size, such as using height squared (SMM/height^2^) (2). In comparison, SMI may be a stronger predictor of sarcopenia in T2DM patients than SMM or LM and should be viewed as a crucial outcome metric in upcoming clinical studies. In conclusion, as one of the most widely used glucose-lowering drugs, although it brings many positive benefits, the potential LM and SMM loss linked to SGLT2 inhibitors-induced weight loss warrants attention. A faster decrease in skeletal muscle and the concomitant rise in the risk of sarcopenia is concerning, especially because those who receive these medicines are frequently already at a higher risk of physical frailty (56). Therefore, we believe it is critical to implement some strategies to protect skeletal muscle while using SGLT2 inhibitors, such as improved nutrition and resistance training (57). It is essential to emphasize that skeletal muscle absolute mass is not the sole component to consider; skeletal muscle strength and physical performance remain critical to performance in the presence of sarcopenia and have an impact on an individual’s quality of life. Nevertheless, only one study evaluating the grip strength of SGLT2 inhibitors *vs* other glucose-lowering medications was included in this meta-analysis, more research into the effects of SGLT2 inhibitors on skeletal muscle strength and athletic performance is required.

Both SGLT2 inhibitors and GLP-1RAs have displayed positive effects on body composition measurements including FM, WC, and LM. Within this meta-analysis, semaglutide has demonstrated superiority in reducing FM and WC when compared to SGLT2 inhibitors, however, the difference was not statistically significant. It is noteworthy that semaglutide exhibited a greater reduction in LM than SGLT2 inhibitors, although there was no statistically significant difference in LM reduction between the two medications. As with other GLP-1RAs, liraglutide also reduces LM in patients with T2DM, although it does not confer any additional advantage over SGLT2 inhibitors in this regard. This meta-analysis includes only two studies that compare the body composition of GLP-1RAs and SGLT2 inhibitors, and the differences between these two drugs are currently unclear. However, the potential negative consequences of LM induced by GLP-1RAs and SGLT2 is warrants attention. Further research is necessary to comprehensively evaluate the differences in body composition changes resulting from the use of these drugs.

The highlight of this meta-analysis was the comprehensive evaluation of the effects of SGLT2 inhibitors on T2DM patients regarding body composition, not only the positive of weight loss, such as BW, BMI, WC, FM, VFA, SFA, but also the negative influence on muscle mass, and consequent increased risk of sarcopenia. However, the followings are this article’s limitations: First, the sample size of the few RCTs that did meet the criteria was small. Second, the majority of these studies only had 24-week follow-up durations, the long-term effects of the SGLT2 inhibitors are also unknown, necessitating ongoing monitoring. Third, due to limited data, only one major indicator of muscle mass was included in this article on sarcopenia; additional RCTs are required to further validate the influence of SGLT2 inhibitors on skeletal muscle strength and physical performance in sarcopenia.

## 5 Conclusion

SGLT2 inhibitors have positive effects on weight loss in T2DM, including BW, BMI, WC, FM, VFA, and SFA, and the SGLT2 inhibitors therapy results in weight loss that is predominantly derived from FM. However, the negative influence on muscle mass is parallel to the reduction in FM and BW, and the consequent increased risk of sarcopenia is noteworthy, especially as patients are already predisposed to physical frailty. Therefore, SGLT2 inhibitors as one of the most widely used hypoglycemic agents should be considered for both benefits on weight loss and harmful muscle reduction of sarcopenia. It is imperative to conduct large-sample and long-term follow-up studies to better understand the risk of sarcopenia and explore strategies for preserving lean mass and improving physical function.

## Data availability statement

The original contributions presented in the study are included in the article/**Supplementary Material**. Further inquiries can be directed to the corresponding author.

## Author contributions

The study was conceived and designed by SZ and DZ. The literature search, data extraction, and statistical analysis were carried out by SZ, ZQ, DS, and YW. SZ write the original draft, while DZ, YW, DS, and DZ severely review and edited it. All authors contributed to the article and approved the submitted version.

## References

[1] Cruz-Jentoft AJ Baeyens JP Bauer JM Boirie Y Cederholm T Landi F . Sarcopenia: European consensus on definition and diagnosis: report of the European working group on sarcopenia in older people. Age Ageing (2010) 39(4):412–23. doi: 10.1093/ageing/afq034 PMC2886201 20392703

[2] Cruz-Jentoft AJ Bahat G Bauer J Boirie Y Bruyère O Cederholm T . Sarcopenia: revised European consensus on definition and diagnosis. Age Ageing (2019) 48(1):16–31. doi: 10.1093/ageing/afy169 30312372 PMC6322506

[3] Scott D de Courten B Ebeling PR . Sarcopenia: a potential cause and consequence of type 2 diabetes in australia’s ageing population? Med J Aust (2016) 205(7):329–33. doi: 10.5694/mja16.00446 27681976

[4] Nowotny K Jung T Höhn A Weber D Grune T . Advanced glycation end products and oxidative stress in type 2 diabetes mellitus. Biomolecules (2015) 5(1):194–222. doi: 10.3390/biom5010194 25786107 PMC4384119

[5] Kim TN Park MS Yang SJ Yoo HJ Kang HJ Song W . Prevalence and determinant factors of sarcopenia in patients with type 2 diabetes: the Korean sarcopenic obesity study (KSOS). Diabetes Care (2010) 33(7):1497–9. doi: 10.2337/dc09-2310 PMC2890348 20413515

[6] Mesinovic J Zengin A De Courten B Ebeling PR Scott D . Sarcopenia and type 2 diabetes mellitus: a bidirectional relationship. Diabetes Metab Syndr Obes (2019) 12:1057–72. doi: 10.2147/dmso.S186600 PMC6630094 31372016

[7] Sun H Saeedi P Karuranga S Pinkepank M Ogurtsova K Duncan BB . IDF diabetes atlas: global, regional and country-level diabetes prevalence estimates for 2021 and projections for 2045. Diabetes Res Clin Pract (2022) 183:109119. doi: 10.1016/j.diabres.2021.109119 34879977 PMC11057359

[8] Tahrani AA Barnett AH Bailey CJ . SGLT inhibitors in management of diabetes. Lancet Diabetes Endocrinol (2013) 1(2):140–51. doi: 10.1016/s2213-8587(13)70050-0 24622320

[9] Tahara A Kurosaki E Yokono M Yamajuku D Kihara R Hayashizaki Y . Effects of SGLT2 selective inhibitor ipragliflozin on hyperglycemia, hyperlipidemia, hepatic steatosis, oxidative stress, inflammation, and obesity in type 2 diabetic mice. Eur J Pharmacol (2013) 715(1-3):246–55. doi: 10.1016/j.ejphar.2013.05.014 23707905

[10] Kelsey MD Nelson AJ Green JB Granger CB Peterson ED McGuire DK . Guidelines for cardiovascular risk reduction in patients with type 2 diabetes: JACC guideline comparison. J Am Coll Cardiol (2022) 79(18):1849–57. doi: 10.1016/j.jacc.2022.02.046 PMC8972581 35512864

[11] Blonde L Stenlöf K Fung A Xie J Canovatchel W Meininger G . Effects of canagliflozin on body weight and body composition in patients with type 2 diabetes over 104 weeks. Postgrad Med (2016) 128(4):371–80. doi: 10.1080/00325481.2016.1169894 27002421

[12] Bode B Stenlöf K Sullivan D Fung A Usiskin K . Efficacy and safety of canagliflozin treatment in older subjects with type 2 diabetes mellitus: a randomized trial. Hosp Pract (1995) (2013) 41(2):72–84. doi: 10.3810/hp.2013.04.1020 23680739

[13] Bolinder J Ljunggren Ö Johansson L Wilding J Langkilde AM Sjöström CD . Dapagliflozin maintains glycaemic control while reducing weight and body fat mass over 2 years in patients with type 2 diabetes mellitus inadequately controlled on metformin. Diabetes Obes Metab (2014) 16(2):159–69. doi: 10.1111/dom.12189 23906445

[14] Cefalu WT Leiter LA Yoon KH Arias P Niskanen L Xie J . Efficacy and safety of canagliflozin versus glimepiride in patients with type 2 diabetes inadequately controlled with metformin (CANTATA-SU): 52 week results from a randomised, double-blind, phase 3 non-inferiority trial. Lancet (2013) 382(9896):941–50. doi: 10.1016/s0140-6736(13)60683-2 23850055

[15] Chehrehgosha H Sohrabi MR Ismail-Beigi F Malek M Reza Babaei M Zamani F . Empagliflozin improves liver steatosis and fibrosis in patients with non-alcoholic fatty liver disease and type 2 diabetes: a randomized, double-blind, placebo-controlled clinical trial. Diabetes Ther (2021) 12(3):843–61. doi: 10.1007/s13300-021-01011-3 PMC7882235 33586120

[16] Fadini GP Bonora BM Zatti G Vitturi N Iori E Marescotti MC . Effects of the SGLT2 inhibitor dapagliflozin on HDL cholesterol, particle size, and cholesterol efflux capacity in patients with type 2 diabetes: a randomized placebo-controlled trial. Cardiovasc Diabetol (2017) 16(1):42. doi: 10.1186/s12933-017-0529-3 28376855 PMC5379610

[17] Han E Lee YH Lee BW Kang ES Cha BS . Ipragliflozin additively ameliorates non-alcoholic fatty liver disease in patients with type 2 diabetes controlled with metformin and pioglitazone: a 24-week randomized controlled trial. J Clin Med (2020) 9(1):259. doi: 10.3390/jcm9010259 31963648 PMC7019437

[18] Horibe K Morino K Miyazawa I Tanaka-Mizuno S Kondo K Sato D . Metabolic changes induced by dapagliflozin, an SGLT2 inhibitor, in Japanese patients with type 2 diabetes treated by oral anti-diabetic agents: a randomized, clinical trial. Diabetes Res Clin Pract (2022) 186:109781. doi: 10.1016/j.diabres.2022.109781 35181350

[19] Hoshika Y Kubota Y Mozawa K Tara S Tokita Y Yodogawa K . Effect of empagliflozin versus placebo on body fluid balance in patients with acute myocardial infarction and type 2 diabetes mellitus: subgroup analysis of the EMBODY trial. J Card Fail (2022) 28(1):56–64. doi: 10.1016/j.cardfail.2021.07.022 34425223

[20] Inoue H Morino K Ugi S Tanaka-Mizuno S Fuse K Miyazawa I . Ipragliflozin, a sodium-glucose cotransporter 2 inhibitor, reduces bodyweight and fat mass, but not muscle mass, in Japanese type 2 diabetes patients treated with insulin: a randomized clinical trial. J Diabetes Investig (2019) 10(4):1012–21. doi: 10.1111/jdi.12985 PMC6626939 30536746

[21] Ito D Shimizu S Inoue K Saito D Yanagisawa M Inukai K . Comparison of ipragliflozin and pioglitazone effects on nonalcoholic fatty liver disease in patients with type 2 diabetes: a randomized, 24-week, open-label, active-controlled trial. Diabetes Care (2017) 40(10):1364–72. doi: 10.2337/dc17-0518 28751548

[22] Javed Z Papageorgiou M Deshmukh H Rigby AS Qamar U Abbas J . Effects of empagliflozin on metabolic parameters in polycystic ovary syndrome: a randomized controlled study. Clin Endocrinol (Oxf) (2019) 90(6):805–13. doi: 10.1111/cen.13968 30866088

[23] Katakami N Mita T Yoshii H Shiraiwa T Yasuda T Okada Y . Tofogliflozin does not delay progression of carotid atherosclerosis in patients with type 2 diabetes: a prospective, randomized, open-label, parallel-group comparative study. Cardiovasc Diabetol (2020) 19(1):110. doi: 10.1186/s12933-020-01079-4 32646498 PMC7350187

[24] Kayano H Koba S Hirano T Matsui T Fukuoka H Tsuijita H . Dapagliflozin influences ventricular hemodynamics and exercise-induced pulmonary hypertension in type 2 diabetes Patients- a randomized controlled trial. Circ J (2020) 84(10):1807–17. doi: 10.1253/circj.CJ-20-0341 32921680

[25] Kinoshita T Shimoda M Nakashima K Fushimi Y Hirata Y Tanabe A . Comparison of the effects of three kinds of glucose-lowering drugs on non-alcoholic fatty liver disease in patients with type 2 diabetes: a randomized, open-label, three-arm, active control study. J Diabetes Investig (2020) 11(6):1612–22. doi: 10.1111/jdi.13279 PMC7610105 32329963

[26] Koshizaka M Ishikawa K Ishibashi R Maezawa Y Sakamoto K Uchida D . Comparing the effects of ipragliflozin versus metformin on visceral fat reduction and metabolic dysfunction in Japanese patients with type 2 diabetes treated with sitagliptin: a prospective, multicentre, open-label, blinded-endpoint, randomized controlled study (PRIME-V study). Diabetes Obes Metab (2019) 21(8):1990–5. doi: 10.1111/dom.13750 PMC6767075 30993861

[27] Latva-Rasku A Honka MJ Kullberg J Mononen N Lehtimäki T Saltevo J . The SGLT2 inhibitor dapagliflozin reduces liver fat but does not affect tissue insulin sensitivity: a randomized, double-blind, placebo-controlled study with 8-week treatment in type 2 diabetes patients. Diabetes Care (2019) 42(5):931–7. doi: 10.2337/dc18-1569 30885955

[28] McCrimmon RJ Catarig AM Frias JP Lausvig NL le Roux CW Thielke D . Effects of once-weekly semaglutide vs once-daily canagliflozin on body composition in type 2 diabetes: a substudy of the SUSTAIN 8 randomised controlled clinical trial. Diabetologia (2020) 63(3):473–85. doi: 10.1007/s00125-019-05065-8 PMC6997246 31897524

[29] Nakaguchi H Kondo Y Kyohara M Konishi H Oiwa K Terauchi Y . Effects of liraglutide and empagliflozin added to insulin therapy in patients with type 2 diabetes: a randomized controlled study. J Diabetes Investig (2020) 11(6):1542–50. doi: 10.1111/jdi.13270 PMC7610130 32279451

[30] Shimizu M Suzuki K Kato K Jojima T Iijima T Murohisa T . Evaluation of the effects of dapagliflozin, a sodium-glucose co-transporter-2 inhibitor, on hepatic steatosis and fibrosis using transient elastography in patients with type 2 diabetes and non-alcoholic fatty liver disease. Diabetes Obes Metab (2019) 21(2):285–92. doi: 10.1111/dom.13520 30178600

[31] Sugiyama S Jinnouchi H Kurinami N Hieshima K Yoshida A Jinnouchi K . Dapagliflozin reduces fat mass without affecting muscle mass in type 2 diabetes. J Atheroscler Thromb (2018) 25(6):467–76. doi: 10.5551/jat.40873 PMC6005223 29225209

[32] Tsurutani Y Nakai K Inoue K Azuma K Mukai S Maruyama S . Comparative study of the effects of ipragliflozin and sitagliptin on multiple metabolic variables in Japanese patients with type 2 diabetes: a multicentre, randomized, prospective, open-label, active-controlled study. Diabetes Obes Metab (2018) 20(11):2675–9. doi: 10.1111/dom.13421 29893003

[33] Wolf VLW Breder I de Carvalho LSF Soares AAS Cintra RM Barreto J . Dapagliflozin increases the lean-to total mass ratio in type 2 diabetes mellitus. Nutr Diabetes (2021) 11(1):17. doi: 10.1038/s41387-021-00160-5 34120150 PMC8197757

[34] Yamakage H Tanaka M Inoue T Odori S Kusakabe T Satoh-Asahara N . Effects of dapagliflozin on the serum levels of fibroblast growth factor 21 and myokines and muscle mass in Japanese patients with type 2 diabetes: a randomized, controlled trial. J Diabetes Investig (2020) 11(3):653–61. doi: 10.1111/jdi.13179 PMC7232283 31721467

[35] Zeng YH Liu SC Lee CC Sun FJ Liu JJ . Effect of empagliflozin versus linagliptin on body composition in Asian patients with type 2 diabetes treated with premixed insulin. Sci Rep (2022) 12(1):17065. doi: 10.1038/s41598-022-21486-9 36224294 PMC9556548

[36] Cohen S Nathan JA Goldberg AL . Muscle wasting in disease: molecular mechanisms and promising therapies. Nat Rev Drug Discovery (2015) 14(1):58–74. doi: 10.1038/nrd4467 25549588

[37] McLeod M Breen L Hamilton DL Philp A . Live strong and prosper: the importance of skeletal muscle strength for healthy ageing. Biogerontology (2016) 17(3):497–510. doi: 10.1007/s10522-015-9631-7 26791164 PMC4889643

[38] Sargeant JA Henson J King JA Yates T Khunti K Davies MJ . A review of the effects of glucagon-like peptide-1 receptor agonists and sodium-glucose cotransporter 2 inhibitors on lean body mass in humans. Endocrinol Metab (Seoul) (2019) 34(3):247–62. doi: 10.3803/EnM.2019.34.3.247 PMC6769337 31565876

[39] Deng MQ Pan Q Xiao XH Guo LX . Interpretations of guideline for the management of diabetes mellitus in the elderly in China (2021 edition). Zhonghua Nei Ke Za Zhi (2021) 60(11):954–9. doi: 10.3760/cma.j.cn112138-20210305-00183 34689515

[40] Davies MJ D’Alessio DA Fradkin J Kernan WN Mathieu C Mingrone G . Management of hyperglycaemia in type 2 diabetes, 2018. a consensus report by the American diabetes association (ADA) and the European association for the study of diabetes (EASD). Diabetologia (2018) 61(12):2461–98. doi: 10.1007/s00125-018-4729-5 30288571

[41] Pereira MJ Eriksson JW . Emerging role of SGLT-2 inhibitors for the treatment of obesity. Drugs (2019) 79(3):219–30. doi: 10.1007/s40265-019-1057-0 PMC6394798 30701480

[42] Palmer SC Tendal B Mustafa RA Vandvik PO Li S Hao Q . Sodium-glucose cotransporter protein-2 (SGLT-2) inhibitors and glucagon-like peptide-1 (GLP-1) receptor agonists for type 2 diabetes: systematic review and network meta-analysis of randomised controlled trials. Bmj (2021) 372:m4573. doi: 10.1136/bmj.m4573 33441402 PMC7804890

[43] Yabe D Nishikino R Kaneko M Iwasaki M Seino Y . Short-term impacts of sodium/glucose co-transporter 2 inhibitors in Japanese clinical practice: considerations for their appropriate use to avoid serious adverse events. Expert Opin Drug Saf (2015) 14(6):795–800. doi: 10.1517/14740338.2015.1034105 25851664

[44] Sasaki T . Sarcopenia, frailty circle and treatment with sodium-glucose cotransporter 2 inhibitors. J Diabetes Investig (2019) 10(2):193–5. doi: 10.1111/jdi.12966 PMC6400153 30369086

[45] Nagai Y Fukuda H Kawanabe S Nakagawa T Ohta A Tanaka Y . Differing effect of the sodium-glucose cotransporter 2 inhibitor ipragliflozin on the decrease of fat mass vs. lean mass in patients with or without metformin therapy. J Clin Med Res (2019) 11(4):297–300. doi: 10.14740/jocmr3785 30937121 PMC6436566

[46] Lundkvist P Sjöström CD Amini S Pereira MJ Johnsson E Eriksson JW . Dapagliflozin once-daily and exenatide once-weekly dual therapy: a 24-week randomized, placebo-controlled, phase II study examining effects on body weight and prediabetes in obese adults without diabetes. Diabetes Obes Metab (2017) 19(1):49–60. doi: 10.1111/dom.12779 27550386 PMC5215525

[47] McGregor RA Cameron-Smith D Poppitt SD . It is not just muscle mass: a review of muscle quality, composition and metabolism during ageing as determinants of muscle function and mobility in later life. Longev Healthspan (2014) 3(1):9. doi: 10.1186/2046-2395-3-9 25520782 PMC4268803

[48] Jayasinghe S Hills AP . Sarcopenia, obesity, and diabetes - the metabolic conundrum trifecta. Diabetes Metab Syndr (2022) 16(11):102656. doi: 10.1016/j.dsx.2022.102656 36356383

[49] Kawata T Iizuka T Iemitsu K Takihata M Takai M Nakajima S . Ipragliflozin improves glycemic control and decreases body fat in patients with type 2 diabetes mellitus. J Clin Med Res (2017) 9(7):586–95. doi: 10.14740/jocmr3038w PMC5458656 28611859

[50] Sneed NM Morrison SA . Body composition methods in adults with type 2 diabetes or at risk for T2D: a clinical review. Curr Diabetes Rep (2021) 21(5):14. doi: 10.1007/s11892-021-01381-9 33730341

[51] Sakurai S Jojima T Iijima T Tomaru T Usui I Aso Y . Empagliflozin decreases the plasma concentration of plasminogen activator inhibitor-1 (PAI-1) in patients with type 2 diabetes: association with improvement of fibrinolysis. J Diabetes Complications (2020) 34(11):107703. doi: 10.1016/j.jdiacomp.2020.107703 32883567

[52] Pan R Zhang Y Wang R Xu Y Ji H Zhao Y . Effect of SGLT-2 inhibitors on body composition in patients with type 2 diabetes mellitus: a meta-analysis of randomized controlled trials. PloS One (2022) 17(12):e0279889. doi: 10.1371/journal.pone.0279889 36584211 PMC9803203

[53] Wong C Yaow CYL Ng CH Chin YH Low YF Lim AYL . Sodium-glucose Co-transporter 2 inhibitors for non-alcoholic fatty liver disease in Asian patients with type 2 diabetes: a meta-analysis. Front Endocrinol (Lausanne) (2020) 11:609135. doi: 10.3389/fendo.2020.609135 33643221 PMC7905212

[54] Bilgin S Duman TT Kurtkulagi O Yilmaz F Aktas G . A case of euglycemic diabetic ketoacidosis due to empagliflozin use in a patient with type 1 diabetes mellitus. J Coll Physicians Surg Pak (2022) 32(7):928–30. doi: 10.29271/jcpsp.2022.07.928 35795946

[55] Bolinder J Ljunggren Ö Kullberg J Johansson L Wilding J Langkilde AM . Effects of dapagliflozin on body weight, total fat mass, and regional adipose tissue distribution in patients with type 2 diabetes mellitus with inadequate glycemic control on metformin. J Clin Endocrinol Metab (2012) 97(3):1020–31. doi: 10.1210/jc.2011-2260 22238392

[56] Janssen I Heymsfield SB Ross R . Low relative skeletal muscle mass (sarcopenia) in older persons is associated with functional impairment and physical disability. J Am Geriatr Soc (2002) 50(5):889–96. doi: 10.1046/j.1532-5415.2002.50216.x 12028177

[57] Villareal DT Chode S Parimi N Sinacore DR Hilton T Armamento-Villareal R . Weight loss, exercise, or both and physical function in obese older adults. N Engl J Med (2011) 364(13):1218–29. doi: 10.1056/NEJMoa1008234 PMC3114602 21449785

