# Supplementary figures and images for: Effect of sodium-glucose transporter 2 inhibitors on sarcopenia in patients with type 2 diabetes mellitus: a systematic review and meta-analysis
Source: Front Endocrinol (Lausanne). 2023 Jul 3;14:1203666. doi: 10.3389/fendo.2023.1203666 (PMC10351980; doi:10.3389/fendo.2023.1203666)

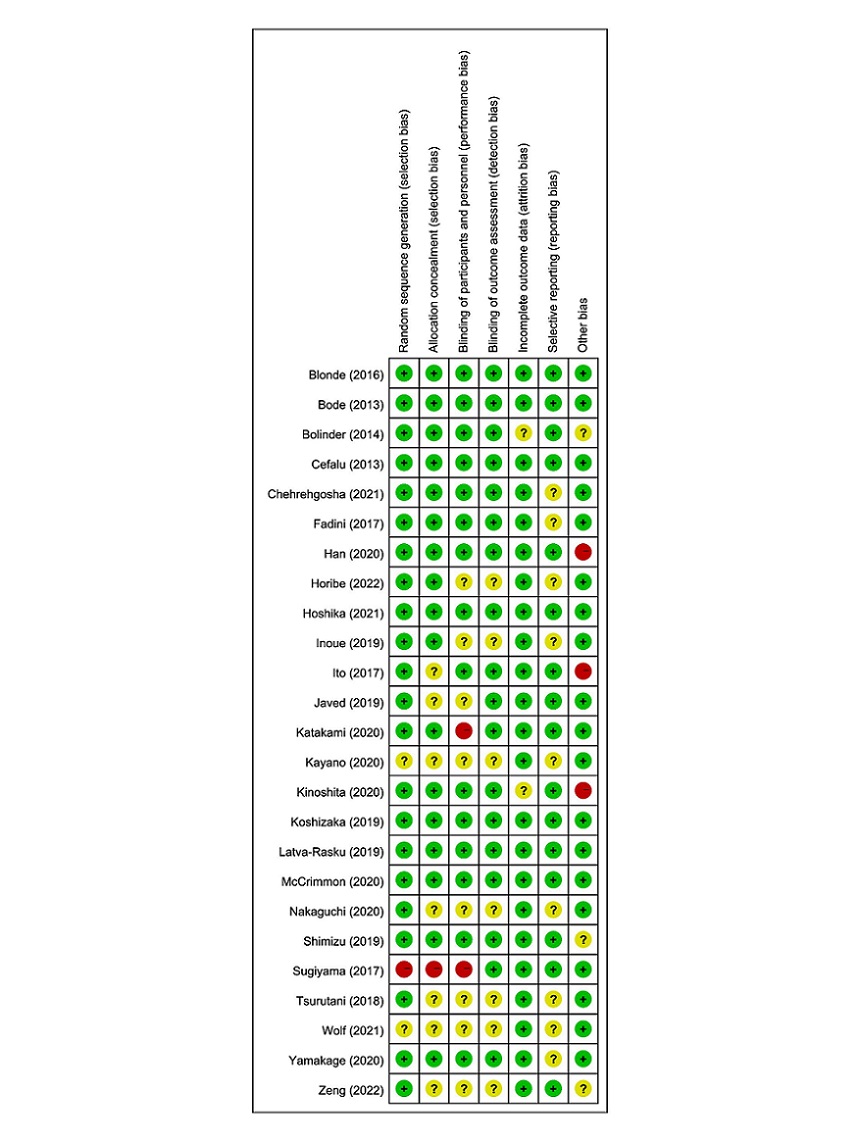

Supplement: Supplementary file 1 [file Image_1.jpeg]
