# Supplementary material for: Effect of sodium-glucose transporter 2 inhibitors on sarcopenia in patients with type 2 diabetes mellitus: a systematic review and meta-analysis
Source: Front Endocrinol (Lausanne). 2023 Jul 3;14:1203666. doi: 10.3389/fendo.2023.1203666 (PMC10351980; doi:10.3389/fendo.2023.1203666)
Supplement: Supplementary file 2 [file Table_1.docx]

Supplementary Material

Effect of sodium-glucose transporter 2 inhibitors on sarcopenia in patients with type 2 diabetes mellitus: a systematic review and meta-analysis

**Sha Zhang, Zhang Qi, Yidong Wang, Danfei Song, Deqiu Zhu***

*** Correspondence:** Deqiu Zhu email：[zdq_0726@163.com](mailto:zdq_0726@163.com)

# Supplementary Table 1 Characteristics of included studies

| Study | Year | Country | Sample size | Age(year) | Comparison(n) | Control(n) | Instrument | Duration |
| --- | --- | --- | --- | --- | --- | --- | --- | --- |
| Blonde L(11) | 2016 | USA | 145 | 63.0 ± 6.0 vs. 64.2 ± 6.4 | Canagliflozin 300mg/d(71) | Glimepiride 6 or 8mg/d(74) | DXA | 104weeks |
| Bode B (12) | 2013 | 17 countries | 145 | 63.4 ± 6.0 vs. 63.2 ± 6.2 | Canagliflozin 300 mg/d(71) | Placebo(74) | DXA | 26weeks |
| Bolinder J (13) | 2014 | 5 countries | 140 | 60.6 ± 8.2 vs. 60.8 ± 6.9 | Dapagliflozin 10 mg/d(69) | Placebo(71) | DXA | 102weeks |
| Cefalu WT (14) | 2013 | 19 countries | 198 | 55.8 ±9.2 vs. 56.3 ±9.0 | Canagliflozin 300 mg/d(102) | Glimepiride 6 or 8mg/d(96) | DXA | 52weeks |
| Chehrehgosha H(15) | 2021 | Iran | 72 | 50.5 ± 8.4 vs. 51.8 ± 7.8 | Empagliflozin 10 mg/d(35) | Placebo(37) | DXA | 24weeks |
| Fadini GP (16) | 2017 | Italy | 31 | 66.3±1.8 vs. 61.0±1.8 | Dapagliflozin 10 mg/d(15) | Placebo(16) | BIA | 12weeks |
| Han E (17) | 2020 | Korea | 44 | 52.5 ± 10.3 vs. 56.7 ± 11.8 | Ipragliflozin50mg/d+Metformin +Pioglitazone(29) | Metformin + Pioglitazone (15) | DXA | 24weeks |
| Horibe K (18) | 2022 | Japan | 50 | 59.7 ± 12.0 vs. 62.3 ± 6.5 | Dapagliflozin 5mg/d+Conventionalmedications(26) | Conventional hypoglycemic treatment(24) | BIA, DXA, MRI | 24weeks |
| Hoshika Y(19) | 2021 | Japan | 51 | 67.5 ± 8.8 vs. 67.0 +10.9 | Empagliflozin10mg /d(22) | Placebo(29) | BIA | 24weeks |
| Inoue H (20) | 2019 | Japan | 46 | 60.5±9.8 vs. 60.8±12.1 | Ipragliflozin 50mg/d+Insulin (22) | Insulin treatment(24) | BIA,DXA,MRI | 24weeks |
| Ito D (21) | 2017 | Japan | 66 | 57.3 ± 12.1 vs. 59.1 + 9.8 | Ipragliflozin50mg/d(32) | Pioglitazone 15–30 mg/d(34) | CT | 24weeks |
| Javed Z (22) | 2019 | UK | 39 | 26.0 ±8.0 vs. 31.5 ±20.0 | Empagliflozin 25 mg/d(19) | Metformin 1500mg/d (20) | BIA | 12weeks |
| Katakami N (23) | 2020 | Japan | 308 | 61.3 ± 9.3 vs. 60.9 ± 9.7 | Tofogliflozin20mg/d(154) | Conventional hypoglycemic treatment(154) | Not available | 104weeks |
| Kayano H (24) | 2020 | Japan | 74 | 69.4±7.1 vs. 66.0±9.5 | Dapagliflozin 5 mg/d(36) | Conventional hypoglycemic treatment(38) | BIA | 24weeks |
| Kinoshita T (25) | 2020 | Japan | 65 | 58.7±1.6 vs. 59.0±1.9 | Dapagliflozin 5 mg/d(32) | Pioglitazone 7.5-15mg/d (33) | DXA | 28weeks |
| Koshizaka M(26) | 2019 | Japan | 98 | 56.6±11.9 vs.  55.7 ± 12.2 | Ipragliflozin 50 mg/d(48) | Metformin 500mg/d (50) | CT | 24weeks |
| Latva-Rasku A (27) | 2019 | Finland | 31 | 62.0± 8.4 vs. 60.0 ±7.4 | Dapagliflozin 10 mg/d(15) | Placebo(16) | MRI | 8weeks |
| McCrimmon RJ (28) | 2020 | 11 countries | 178 | 58.6±10.1 vs. 57.8±9.9 | Canagliflozin 300 mg/d(90) | Semaglutide 1.0mg/d(88) | DXA | 52weeks |
| Nakaguchi H (29) | 2020 | Japan | 61 | 66.3 ± 9.5 vs. 67.2 ± 9.0 | Empagliflozin 10 mg/d(31) | Liraglutide 0.9mg/d(30) | DXA | 24weeks |
| Shimizu M(30) | 2019 | Japan | 57 | 56.2±11.5 vs. 57.1±13.8 | Dapagliflozin 5mg/d(33) | Conventional hypoglycemic treatment(24) | BIA | 24weeks |
| Sugiyama S (31) | 2017 | Japan | 50 | 55.6±7.4 vs. 56.7±7.9 | Dapagliflozin5 mg/d(28) | Conventional hypoglycemic treatment(22) | BIA | 24weeks |
| Tsurutani Y (32) | 2018 | Japan | 101 | 53.5±11.7 vs.  54.0 ± 10.7 | Ipragliflozin 50 mg/d(52) | Sitagliptin once daily(49) | BIA | 12weeks |
| Wolf VLW (33) | 2021 | Brazil | 89 | 58.0±7.0 vs. 58.0±7.0 | Dapagliflozin 10mg/d(44) | Glibenclamide 5mg/d(45) | DXA | 12weeks |
| Yamakage H (34) | 2020 | Japan | 50 | 58.4 ±13.0 vs. 60.7 ± 11.9 | Dapagliflozin5mg/d+Conventional medications(26) | Conventional hypoglycemic treatment(24) | BIA | 24weeks |
| Zeng YH (35) | 2022 | Taiwan | 97 | 58.9 ± 9.9 vs. 58.7 ± 10.2 | Empagliflflozin 25mg/d +Premixed insulin(46) | Linagliptin 5mg/d+Premixed insulin(51) | BIA | 24weeks |
